# The Children's Bill

**Published:** 1908-03-14

**Authors:** 


### The Hospital

A Journal of

The Medical Sciences and Hospital Administration.

New Series. No. 52, Vol. II. [No. 1124, Vol. XLIII.] SATURDAY, MARCH 14, 1908.

### THE CHILDREN'S BILL.

It is one of the anomalies of our political life that many measures lag behind the acknowledged sanction of public opinion, while others are placed upon the Statute Book in an almost entire absence of popular demand. The Children's Bill is one of those measures for which public demand has run well ahead of legislative enactment. The task of the Government in framing a Bill which, while excluding controversial topics, should codify, amend, and extend the law for the protection of children must have been much greater than it has proved had it not been for the wide recognition of the fact that such a measure was urgently called for. We welcome the Government proposals introduced by Mr. Samuel as an instalment of a more rational system for securing child culture and protection. The Children's Bill is divided into five parts:—

1. Protection of Infant Life.
2. Prevention of Cruelty to Children.
3. Measures against Juvenile Smoking.
4. Consolidation of the Law relating to Reformatories and Industrial Schools.
5. Establishment of Children's Courts.

Part 1 of the Bill deals with extremely important statutory provisions. It embodies the Infant Life Protection Act of 1897, which has been found defective in many respects, and it is purposed to amend some of the less controversial of these defects. One of the more disputable defects has been referred to a Select Committee, which will report, it is hoped, in time for its recommendations to be embodied in the Bill at its later stages. This relates to the omission in the 1897 Act to provide for the inspection of those persons who receive a single nurse child in consideration, not of a lump sum, but of periodical payments. This exemption undoubtedly leads to most serious evils not only in the case of unscrupulous baby farmers on a small scale, but also in the case of well-intentioned foster-mothers who receive children whose care they are utterly unfitted to undertake.

We are further strongly of opinion that the administration of this Act, as embodied in Part 1 of the Bill, relating to Infant Life Protection, should wherever possible devolve upon the local authorities, and not upon the Boards of Guardians, as for the most part is now the case. The relieving officer is not a suitable inspector for the purposes of this Act. So many urban authorities are now provided with lady health visitors and lady sanitary inspectors who are ideal inspectors for this purpose that we hope it will be found possible where such officers have been appointed to make them inspectors under the Act. Baby farms would in this way come under the supervision of the medical officer of health, who is the officer most intimately concerned in the prevention of infantile mortality. It has been proved that the high mortality among illegitimate children, who largely constitute the *clientèle* of the baby farmer, is due to preventable conditions which call for the intervention of the nurse and the medical man rather than of the relieving officer.

Part 2 of the Bill deals with an equally important province of protective legislation. It re-enacts the Prevention of Cruelty to Children Act of 1894 and amends and strengthens the law so as to facilitate the action of the societies doing such valuable work under the Act. In particular it will make "overlaying" a penal offence for which it will provide a light penalty. In Germany and other countries the practice of taking the baby to sleep in the same bed with the mother, which is the cause of overlying, is a penal offence, and we think this the wiser provision. It may be urged that it is not possible to discover breaches of this law save when they lead to a fatal issue. Even so, this would be sufficient. The weight of the law would be on the side of hygienic practice, and the punishment would fall upon offenders when death from suffocation due to overlying occurred, not because of an accident incidental to the practice, but because of the practice which itself is harmful and dangerous. We are glad to note that this part of the Bill makes punishable also the leaving of children in rooms with unguarded fires. Hundreds of children every year are burned or scalded to death due to the unpardonable negligence of their parents.

The third part of the Bill deals with juvenile smoking. The evils of this growing practice have for long been pointed out by medical men, and the reports of the Inter-Departmental Committee on Physical Deterioration and of a Committee of the House of Lords are at one on the need for legislative measures such as are proposed in the Bill. The proposals of the

Government in regard to this practice seem wise. The sale of cigarettes and cigarette paper to children under 16 years of age will be prohibited by law, while the police and authorised persons will have power to confiscate tobacco found on boys smoking in streets and public places.

Parts 4 and 5 of the Bill propose remedies for what has long been recognised as a scandal in our criminal procedure against juvenile offenders. The moral contamination which follows from treating children's offences in the same manner as those of the hardened criminal, and from permitting old offenders to mix with boys and girls already in some cases predisposed to a criminal career has for long been recognised as a potent circumstance in the development of criminal propensities. Perhaps the most useful amendment in this connection is that which proposes to establish throughout the country children's courts in which children's cases should be heard at a separate time, or in a separate room from the courts which are held for adult cases. It is further proposed in London to appoint a children's magistrate to visit in turn a circuit of courts. These courts will have as their aim the rescue rather than the punishment of children, and will endeavour to bring home to the parents their responsibility for the wrong-doing of the child. We have ourselves heard in a police court a mother recommending her boy to "do time" when the choice was offered him by a magistrate to go to a disciplinary home for a longer period than that for which he would be committed to prison. And the child went to prison and the mother went scot-free. This is an illustration of some of the defects which the Bill will do something to remove. It has been said that we make criminals by the very steps we take to prevent crime, and this is unquestionably true of our treatment of juvenile offenders. The commitment of children to common gaols, there to mix with adult criminals and familiarise themselves with those penalties of imprisonment which should retain the vague terrors of the unknown, is barbarous and irrational. The Bill marks a great advance in the civic status of the child. The natural developmental differences between the child and the adult have become the subject of forensic distinction, and in the prevention as distinguished from the punishment of crime is established an enlightened precedent in legislative prophylaxis.

